# The timing of drain removal in parotidectomies: outcomes of removal at 4 h post-operatively and a Canadian survey of practice patterns

**DOI:** 10.1186/s40463-023-00665-2

**Published:** 2023-09-13

**Authors:** Alice Q. Liu, Oleksandr Butskiy, Veronique Wan Fook Cheung, Donald W. Anderson

**Affiliations:** 1 Division of Otolaryngology-Head and Neck Surgery, Diamond Health Care Centre, University of British Columbia, 2775 Laurel St, 4th Floor ENT Clinic, Vancouver, BC V5Z 1M9 Canada; 2 Division of Otolaryngology, Michigan State University, Grand Rapids, MI USA

**Keywords:** Parotidectomies, Outpatient surgery, Canadian, Otolaryngology, Clinical practice

## Abstract

**Background:**

The post-operative management of parotidectomies is highly provider dependent. No guidelines are currently available for timing of parotid drain removal. This study aimed to assess: (1) outcomes and complications after early drain removal (< 4 h, post-operative day [POD] 0) versus late drain removal (POD ≥ 1); (2) current Canadian provider practices.

**Methods:**

A single surgeons ten-year parotidectomy practice was reviewed, spanning his practice change from routine POD ≥ 1 drain removal to POD 0 removal, with extraction of patient demographic, disease, and complication variables. An anonymous, cross-sectional survey on parotid drain practices was distributed to Canadian Society of Otolaryngology-Head and Neck Surgery members. Descriptive statistics, Wilcoxon Rank Sum, and unpaired student’s t-tests were calculated.

**Results:**

In total, 526 patients were included and 44.7% (235/526) had drains removed POD 0. There was no significant difference in hematoma or seroma rates between the POD 0 and POD ≥ 1 drain removal cohorts. The national survey on parotid drain management had 176 responses. The majority (67.9%) reported routinely using drains after parotidectomy and 62.8% reported using a drain output based criteria for removal. The most common cut-off output was 30 ml in 24 h (range 5–70 ml).

**Conclusion:**

There was no difference in hematoma or seroma rates for patients with parotid drains removed on POD 0 versus POD ≥ 1. Our national survey found significant variation in Canadian parotidectomy drain removal practices, which may be an area that can be further assessed to minimize hospital resources and improve patient care.

**Graphic abstract:**

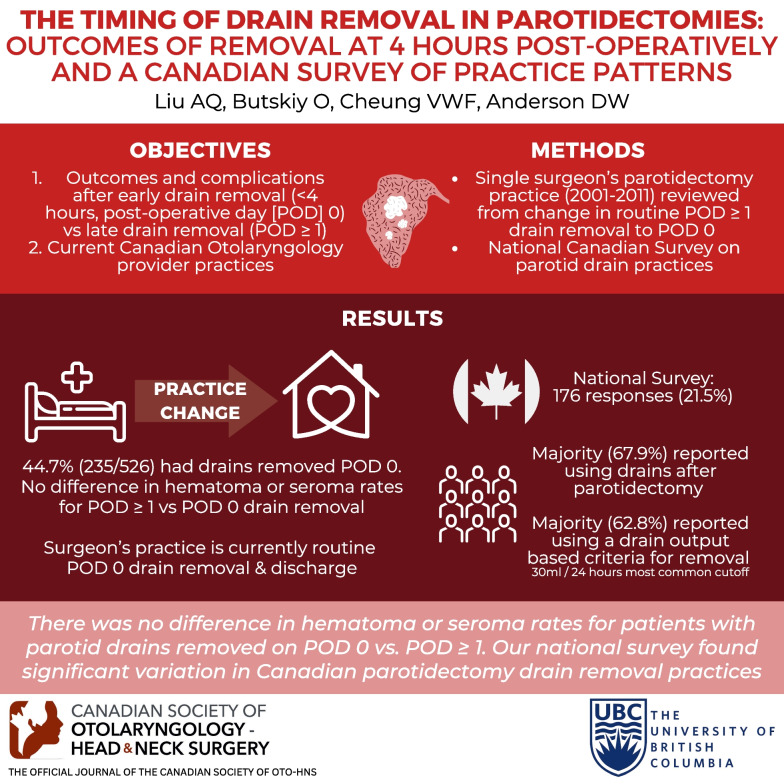

## Background

Parotidectomies are a common surgical procedure performed in otolaryngology. It is estimated that the incidence of salivary malignancy is 0.5–3/100,000 people per year in North America [1, 2]. However, parotidectomies can also be performed for malignant, benign, or diagnostic purposes.

There are currently no consensus statements or clinical practice guidelines in North America surrounding post-operative parotid drain management. Historically, parotid drains have been used to close dead space, remove drainage, and are thought to decrease seroma and hematoma formation [3]. Yet, post-operative parotid drain care is usually provider dependent. At our institution, one surgeon has changed their practice from previously admitting patients after parotidectomies to monitor their drain output to consistently removing parotid drains while the patient is in recovery prior to same day discharge. This was thought to be safe and practical, allowing parotidectomies to be an outpatient day procedure that did not require admission or an additional office visit for drain removal.

There is little published literature on post-operative drain management in parotidectomies. A study of otolaryngology-head and neck surgery graduates in Ontario, Canada noted recent graduates frequently performed parotidectomies once in practice [4]. Yet, it is still largely undefined what post-operative parotid drain management for practicing North American surgeons may be.

The primary objectives of this study were to: (1) analyze the outcomes of early drain removal (at four hours, post-operative day (POD) 0) compared to late drain removal (POD ≥ 1); (2) assess the variabilities in the timing of parotid drain removal post-operatively on a national scale.

## Methods

### Patients

Ethics approval for the chart review was obtained through our institution’s clinical research ethics board (H11-01433). Consecutive patients undergoing parotidectomy between January 1st, 2001 and June 15th, 2011 were identified through the senior surgeon’s electronic billing record and charts retrospectively reviewed. This time frame was chosen to include when the surgeon shifted their practice from POD ≥ 1 removal to same-day drain removal (POD 0) after parotidectomies. Practice shifted from routine POD ≥ 1 removal pre-January 2007 to routine POD 0 drain removal post-January 2007 as this was thought a safe and efficient use of hospital resources. There was no volume cut-off for POD 0 drain removal. However, if a patient were a planned admission secondary to surgical, anesthetic, or social concerns after the practice shift to POD 0 drain removal, drains were often not removed until POD ≥ 1 as the patient was not meant for immediate discharge and admission was not for drain monitoring. Exclusion criteria included concurrent procedures such as neck dissections or large ablative composite resections.

Patients received a standard Blair incision and all parotids were resected by the senior author. Standard closure was done primarily with a deep muscular layer re-approximated and then the skin closed with subcuticular absorbable sutures. A 7 mm flat closed drain was placed outside of the incision in the neck. During the time frame of the retrospective review, the senior surgeon changed practice to routinely discharging patients with drain removal at four hours post-operatively (POD 0). Previously, standard practice was to admit the patient after parotidectomy until the drain output decreased to approximately less than 30 mL in 24 h. No routine pre-operative or post-operative antibiotics were given unless there was felt to be concerns during the procedure.

### Data extraction

Electronic medical records and patients charts were reviewed. Data collected included the patients’ age, sex, extent of parotidectomy, side of surgery, length of hospital stay, timing of drain removal, complications such as hematoma, seroma, infection, or abscess formation, and re-admissions to hospital due to their parotid surgery.

Descriptive statistics were used to assess patient demographic factors. Continuous data was analyzed using an unpaired t-test. Categorical data was analyzed using Fischer’s Exact test. Statistical analysis was done with Excel and P defined as < 0.05.

### National survey

An anonymous, online, cross-sectional survey was sent to current members of the Canadian Society of Otolaryngology-Head and Neck Surgery (CSO-HNS) through email lists maintained by their respective organizations. The survey was open for three months from October 2019 to February 2020. Consent was obtained from participants prior to survey initiation.

The survey structure consisted of six questions, created by the authors to target assessment of parotidectomy practice. This included assessing the providers subspecialty, number of parotidectomies performed per year, drain use, factors affecting drainage use, type of drain, and criteria for drain removal. Residents and pediatric subspecialists were excluded from the final analysis. If providers stated they did not perform parotidectomy, they were also excluded. Descriptive statistics were calculated of the complete responses that remained.

## Results

### Patients

A total of 629 consecutive patients were identified on chart review. After exclusion criteria were applied, 388 patients who underwent superficial parotidectomies and 138 patients who underwent deep/complete parotidectomies were included for a total of 526 patients. Please refer to Table 1 for patient demographics. Drains were routinely removed four hours post operatively on POD 0 in 44.7% (235/526) of patients compared to 55.3% (291/526) of patients who had drains removed per the senior surgeon’s previous practice on POD ≥ 1. No patients after the change in practice to POD 0 drain removal were then admitted from post-anesthetic recovery due to drain concerns. The average age for the POD 0 cohort was statistically younger than the POD1 cohort (50 vs. 56 years, p < 0.001).

**Table 1:** Patient demographics and length of hospitalization

	Drain removed 4 h postoperatively (POD 0)	Drain removed ≥ 1 day postoperatively (POD ≥ 1)	p-value
N (%)	235 (45%)	291 (55%)	
Av. Age (yr) ± SD	50 ± 15	56 ± 17	< 0.001*
% Male	50%	54%	0.38
# of Superficial parotidectomies	180/235 (77%)	208/291 (71%)	0.20
# of complete parotidectomies (%)	55/235 (23%)	83/291 (29%)	0.20
Malignant pathology (%)	23/235 (1.0%)	29/291 (1.0%)	1
Average length of hospital stay (nights)	0.04 ± 0.21	1.08 ± 0.36	< 0.001*

*P value < 0.05

### Parotid outcomes

The overall hematoma rate was 2.3% (12/526). Of these twelve patients, four developed hematomas with their drain still in place, prior to removal, less than 4 h post-operatively. There were thus excluded from the hematoma rates analysis (Table 2). Of the eight patients analyzed, 2.6% (6/235) of patients developed hematomas in the POD 0 cohort compared to 0.7% (2/291) of patients in the POD ≥ 1 cohort. There was no significant difference in hematoma formation between the POD 0 or POD ≥ 1 cohorts (p = 0.15). There was also no significant difference in hematoma formation between malignant or non-malignant cases or between deep/complete parotidectomy and superficial parotidectomy.

**Table 2:** Complication rates and length of hospital stay according to timing of drain removal

	Drain removed POD0, at four hours (N:235)	POD ≥ 1 (N:291)	P-value
Hematoma rate (%)	2.6 (6/235)	0.7 (2/291)	0.15
% superficial parotidectomies	1.7 (3/180)	1.0 (2/208)	0.67
% complete parotidectomies	5.5 (3/55)	0 (0/83)	0.06
Length of stay for patients with hematomas (days)	1.0 ± 0.6	1.5 ± 0.7	0.38
Seroma rate (%)	2.1 (5/235)	1.0 (3/291)	0.48
% superficial parotidectomies	2.2 (4/180)	1.0 (2/208)	0.42
% complete parotidectomies	1.8 (1/55)	1.2 (1/83)	1

Upon chart review, patients with hematoma formation had a history of anticoagulation medication use such as aspirin, naproxen, warfarin and heparin, uncontrolled hypertension and/or Valsalva or vigorous straining postoperatively (Table 3).

**Table 3:** Demographics of patient who developed hematoma

Case #	Timing of hematoma	Drain present	Circumstance of developing hematoma	Pathology
1	POD 0	Yes	Coumadin stopped 3–4 days ago and on LMWH until surgery	Pleomorphic adenoma and Warthin’s tumor
2	POD 0	Yes	Coughing fit post extubation	Lymphoepithelial cyst
3	POD 0	Yes	Hypertensive, requiring labetalol	Oncocytoma
4	POD 0	Yes	On Aspirin up to the day of the surgery	Pleomorphic adenoma
5	POD 0	No	15 min post drain removal	Pleomorphic Adenoma
6	POD 0	No	Going to bathroom prior to discharge, after drain removed	Metastatic Squamous Cell CA
7	POD 0	No	After drained removed while eating on POD 0	Myeloepithelioma
8	POD 1	No	On Naproxen, post drain removal POD 1	Hodgkin’s
9	POD 1	No	Query recurrent acinic cell CA; post drain removal POD 1	No malignancy
10	POD 1	No	Drain removed POD 0	Lipoma
11	POD 3	No	On Naproxen; drain removed on POD 0	Warthin’s tumor
12	POD 5	No	Drain removed POD 0	Pleomorphic adenoma

The overall seroma rate was 1.5% (8/536), with 2.1% (5/235) of patients developed seromas in the POD 0 cohort compared to 1.0% (3/291) of patients in the POD ≥ 1 cohort. There was no significant different in seroma formation between the POD 0 or POD ≥ 1 cohorts (p = 0.48). There was also no significant difference in seroma formation between malignant or non-malignant cases or between deep/complete parotidectomy and superficial parotidectomy.

In regards to infection, 11 of the 526 patients received prophylactic antibiotics pre-operatively or at the time of surgery who were excluded from the analysis. It was found that 9.7% (50/515) of patients received antibiotics post-operatively for symptoms ranging from increased wound pain, swelling and redness, to abscess formation. There was no significant difference in the number of patients who received antibiotics or the rate of abscess formation between the cohort who had their drains removed POD 0 compared to POD ≥ 1.

### National survey

**Fig. 1:**
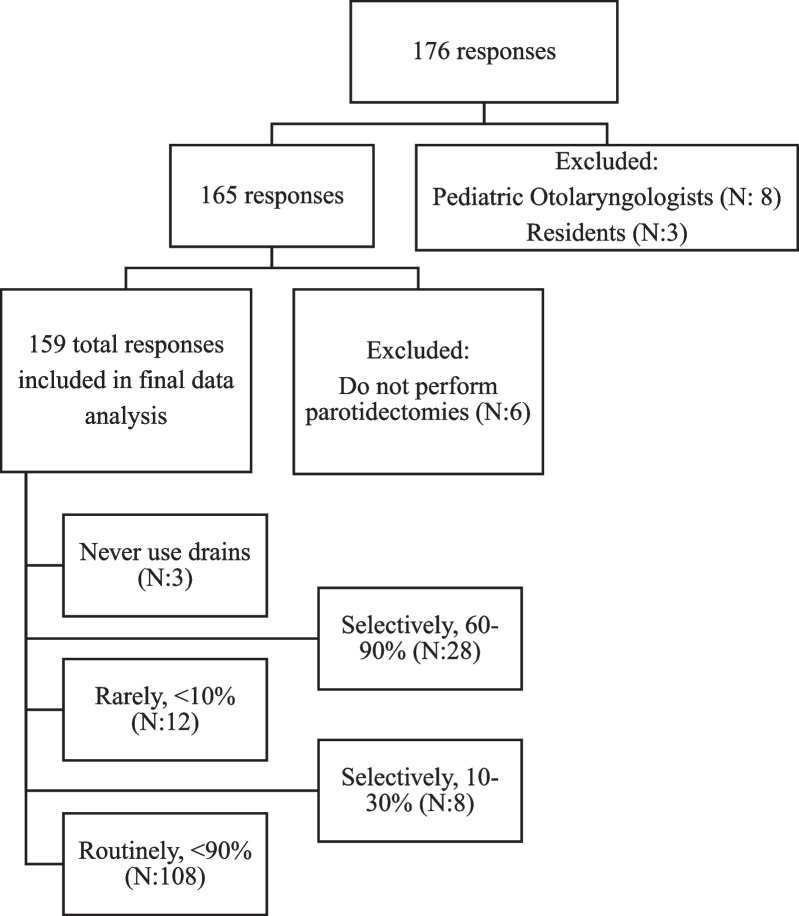
Attrition and response rate of patients who responded to survey on use of surgical drains following isolated parotid surgery. Survey responses were collected from October 2019 to February 2020

**Fig. 2:**
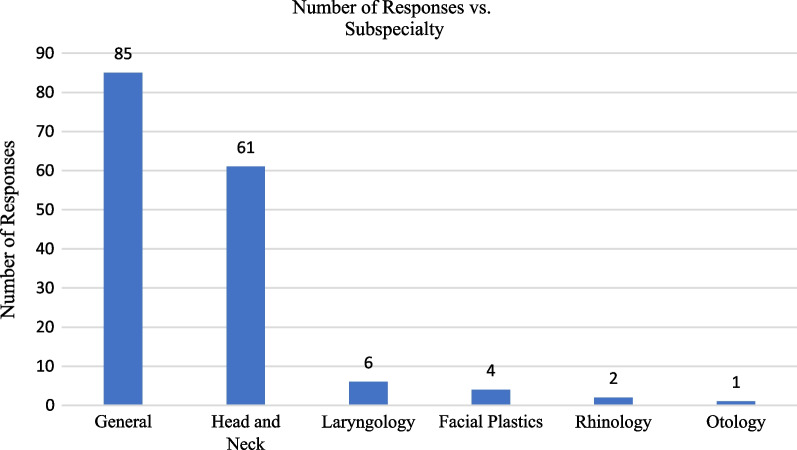
Participants self-reported subspecialty

In total, 176 responses were recorded from approximately 817 members of the CSO-HNS mailing list. After exclusion, 159 providers responses were included in the analysis. The response and attrition rate are shown in Fig. 1. The majority of responses were from general otolaryngologists (N: 85, 54.5%), followed by head and neck surgeons (N: 61, 39.1%) (Fig. 2). Of those that responded, just under half stated they performed between 10 and 30 parotidectomies a year (N: 76, 47.8%). This was followed by providers stating they performed less than 10 parotidectomies annually (35.2%), and then those that stated they performed more than 30 parotidectomies annually (N:27, 17.0%).

**Fig. 3:**
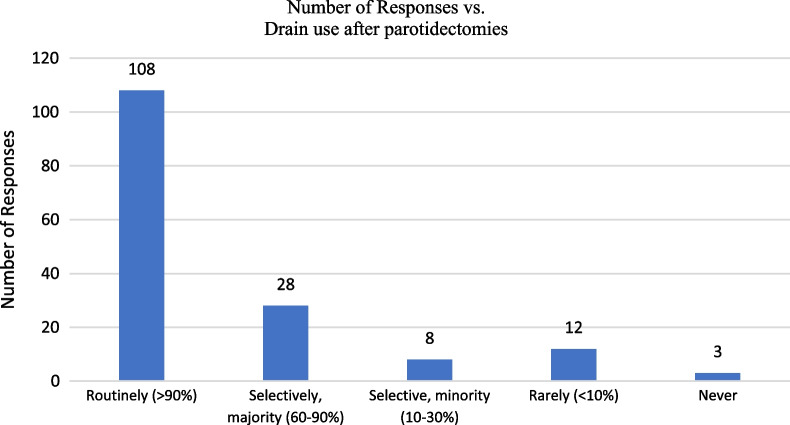
Participant reported frequency of drain use

The majority of providers routinely used a drain (N:108) (Fig. 3), and closed drains were favored over open drains (N: 146 vs. N: 10 respectively, *X*^2^ < 0.001) Respondents stated they were most likely to use a drain output based criteria for removal (62.8%), followed by a time based criteria (28.8%), both drain output and time criteria (6.4%), or other factors (1.9%).

Of those using a drain output based criteria for drain removal, most stated their criteria was ≤ 30 ccs in 24 h or ≤ 20 ccs in 8 h (Table 4). Six providers did not specify the exact output they used as criteria. Of those using a time based criteria for drain removal, the majority stated they remove their drains on POD1 (66.1%) followed by POD2 (12.5%), with POD0 and POD > 3 removal being at the same rate (7.1%). Four providers did not specify the exact time they removed their drains using a time based criteria.

**Table 4:** The drain output criteria for removal from providers who stated they considered drain output prior to removal of drains after parotidectomies (N = 109, 98 who used solely drain output based criteria, 11 who used drain output and time based criteria)

Drain output	Time frames		
	24 h	12 h	8 h
≤ 10 ccs (%)	4 (3.7)	0	3 (2.8)
≤ 20 ccs (%)	12 (11.0)	2 (1.8)	8 (7.3)
≤ 30 ccs (%)	54 (49.5)	1 (0.9)	1 (0.9)
≤ 40 ccs (%)	3 (2.8)	1 (0.9)	1 (0.9)
≤ 50 ccs (%)	11 (10.1)	1 (0.9)	0
> 50 ccs (%)	1 (0.9)	0	0
Did not specify	6 (5.5)		

## Discussion

To our knowledge, this is the largest study assessing a single surgeons practice with timing of parotid drain removal, and the first study to survey Canadian otolaryngologists about their practices surrounding parotid drain management.

### Drain removal at POD0 versus POD ≥ 1

There is no current clear consensus or North American guideline on parotid drain management. Outpatient parotidectomies have been described since 1991 [5], but provider variability in post-operative management remains and some patients stay in hospital until the drain is removed. This differs from other procedures in otolaryngology, such as thyroidectomies, where same-day discharges are recommended by governing bodies as safe in the correct patient population [6]. Factors that have been previously attributed to the success of outpatient thyroidectomies in otolaryngology include lack of significant patient co-morbidities, patient proximity to hospital, and availability of social support [7].

Based on our ten-year retrospective review, the only significant demographic difference between the patients who had their drains removed four hours post-operatively compared to the patients who had their drains removed POD ≥ 1 was their age (50 vs. 56 years respectively, p < 0.001) (Table 1). This may be due to older patients generally being more comorbid, which may require admission to hospital secondary to surgical, anesthetic, or social concerns. If patients were already scheduled to be admitted after the practice change to POD 0 drain removal, the patient’s drain would be left for removal on POD ≥ 1 as they would still be in hospital regardless. Other studies have also found patients who were kept in-hospital after parotidectomy were significantly older [8]. Chen et al.’s study on post-operative drain output after parotidectomies found that body weight was the only patient demographic factor significantly associated with increased post operative drain output [9].

In our 526 patients, the patients who had their drains removed POD ≥ 1 did have longer lengths of stay, as expected. There was no difference in development of a hematoma or seroma in early drain removal at four hours post-operatively (POD 0) compared to drain removal POD ≥ 1. Although there was a trend towards a higher hematoma formation rate in the POD 0 cohort than the POD ≥ 1 cohort, the limited power of this 10-year retrospective study could not bring out any statistically significant difference in hematoma complication rates (p = 0.15).We also did not find any statistically significant difference in seroma rates between the two cohorts (Table 3).

Roh and colleagues demonstrated that parotid gland salivary flow rate was higher after partial parotidectomy compared to conventional parotidectomies [10]. The amount of saliva and potential for sialocele formation are considerations providers must consider when debating drain management after parotidectomies [11]. In our study, there was no difference in hematomas or seromas when assessing superficial vs. deep/complete parotidectomies. Our hematoma and seroma rates for patients with same-day drain removal (2.6% and 2.1% respectively) are similar to other reported values in the literature of 3.8–6.1% and 2–10% [3, 12–14].

Being aware of variables such as anticoagulation use, coagulopathy, and uncontrolled hypertension (Table 3) that may play a role in parotid hematoma development could help providers choose which patients need longer drain management. We found no significant difference in hematoma or seromas based off malignant versus benign pathology. Molfe and Urquhart, in contrast, examined 69 patients who underwent superficial parotidectomies and found malignant pathology was significantly associated with increased post-operative drain output [3].

An analysis of the American College of Surgeons National Surgical Quality Improvement Program reported an overall total complication rate of 5.3% after parotidectomy [15]. Our infection rate of 9.7% was higher than the rates reported of 3.8–5.4% [12, 16] but there was no significant difference when comparing the infection rates between the POD 0 and POD ≥ 1 cohort. We defined infection as patients who received antibiotics, and this broad definition as well as the lack of perioperative prophylactic antibiotics may contribute to our higher infection rate.

Given the low incidence of hematoma formation (2.6%) and the shortened hospital stay in patients who have drains removed at four hours (0.40 days vs. 1.08 days, p < 0.001), the senior surgeon has chosen to continue the practice of removing drains prior to same day discharge.

### National survey

Overall, our survey response rate was approximately 21.5%, with 176 responses. There is high variability in online surveys of surgeons, with reports ranging from 9 to 80% [17]. Our response rate is comparable to recent Canadian national surveys in Otolaryngology that range from 22 to 30% [18–20]. To our knowledge, this is the first North American examination of practice in drain management after parotidectomy.

Of our respondents, just under half (47.8%) performed between 10 and 30 parotidectomies annually. The majority of responses (62.8%) favored closed drains, which is also the preference of our senior surgeon. Of those using a drain output based criteria for drain removal, most stated their criteria was ≤ 30 ccs in 24 h or ≤ 20 ccs in 8 h (Table 4). For those that favored a time based criteria for drain removal, the most common response was removal on POD1 (66.1%) followed by POD2 (12.5%). Comparing these values to other reports in the literature, there are studies that both report higher and lower drain output cut-offs for removal. Harris and colleagues assessed the timing of drain removals after parotidectomies and found a safe profile in removal when the volume was less than or equal to 50 mL after 24 h [21]. In comparison, another study removed drains when the output was less than 5 mL after 8 h [3].

There were three participants who stated they never used drains and twelve who stated they ‘rarely used drains (< 10%)’. A large recent study from Denmark of 205 patients undergoing superficial parotidectomy compared those with drain outputs less than 25 mL compared to those with more than 25 mL in 24 h [22]. The authors reported that 7.3% of patients developed seromas or hematomas in spite of drain placement and that the choice of placing a drain was not significantly associated with drain output.

### Outpatient parotidectomies

Medicine has moved towards less invasive procedures and minimizing hospital stays to improve patient outcomes and curb accelerating healthcare costs. A retrospective review of 42 drainless patients compared to 49 patients with drains after parotidectomy supports the argument for early discharges. The authors found a trend towards more seromas in the patients without drains, but this was not significant (p = 0.298) [23]. None of their drainless patients required readmission or experienced major complications. Our study, in comparison, also assessed the practice of minimizing healthcare resources by reducing admissions and routinely removing drains POD 0 with same day discharge. The seroma and hematoma rate in our patients included also trended higher in the POD 0 group than the POD ≥ 1 group, but this was also not significant.

Recent systematic reviews also support the safety of outpatient parotidectomies, with similar complication and re-admission rates when compared to inpatient parotidectomies [24, 25]. Lee et al. retrospectively examined 238 patients who underwent superficial parotidectomy and found no difference in complication rates, return to the emergency department, or readmission within 30 days after outpatient vs. inpatient post-operative stays [8]. Their cut-off for drain removal was < 30 mL in 24 h. Similar to our study, the found that the inpatient cohort who stayed overnight were statistically older than the outpatient cohort.

### Limitations

Limitations of this study include the inherent bias associated with a retrospective review and the selection bias of providers who choose to answer the survey on drain management. Our cohort included heterogenous pathologies (benign and malignant cases), although there were no differences in hematoma or seromas based off pathology. Unfortunately, we were also not able to assess the exact amount of the parotid tissue removed, which may have made a difference when assessing drain usage or post-operative complication rates. Furthermore, the authors do acknowledge that this study examines a single surgeons practice and that the results may not be generalizable. Some practitioners discharge their patients with drains in place for removal at in-office follow-up, which would still decrease hospital stays. However, these providers then do require follow-up, which may be inconvenient to patients. Lastly, the national survey data focuses on providers current practice but did not specifically ask the reasoning of providers, which would have provided additional information. Our response rate for the survey data was low (21%), but comparable to other recent national surveys of Canadian Otolaryngologists (22–30%) [18–20].

## Conclusion

Our study of a large retrospective cohort found no difference in hematomas or seroma rates after outpatient parotidectomies with drain removal at four-hours post-operatively compared to in-patient parotidectomies with delayed drain removal. The authors found that most Canadian providers used a closed drain, and chose drain output criteria for removal as either < 30 mL in 24 h or < 20 mL in 8 h. We would advocate that in the correct patient population, outpatient parotidectomies could be more widely accepted as they can contribute to less hospital admissions and patient benefits.

## Data Availability

The datasets used and/or analysed during the current study are available from the corresponding author on reasonable request.

## Abbreviations

POD: Post-operative day
CSO-HNS: Canadian Society of Otolaryngology-Head and Neck Surgery
