# Correction: The timing of drain removal in parotidectomies: outcomes of removal at 4 h post-operatively and a Canadian survey of practice patterns

**DOI:** 10.1186/s40463-023-00678-x

**Published:** 2023-10-26

**Authors:** Alice Q. Liu, Oleksandr Butskiy, Veronique Wan Fook Cheung, Donald W. Anderson

**Affiliations:** 1 Division of Otolaryngology-Head and Neck Surgery, Diamond Health Care Centre, University of British Columbia, 2775 Laurel St, 4Th Floor ENT Clinic, Vancouver, BC V5Z 1M9 Canada; 2 Division of Otolaryngology, Michigan State University, Grand Rapids, MI USA

## Correction: Journal of Otolaryngology—Head & Neck Surgery (2023) 52:60 https://doi.org/10.1186/s40463-023-00665-2

The original article [1] contains an error in Fig. 1. In the lower-left box, it should instead state: ‘ > 90%’.
